# Adherence to the Mediterranean Diet and Circulating Levels of Sirtuin 4 in Obese Patients: A Novel Association

**DOI:** 10.1155/2017/6101254

**Published:** 2017-06-15

**Authors:** Luigi Barrea, Giovanni Tarantino, Carolina Di Somma, Giovanna Muscogiuri, Paolo Emidio Macchia, Andrea Falco, Annamaria Colao, Silvia Savastano

**Affiliations:** ^1^I.O.S. & COLEMAN Srl, Acerra, 80011 Naples, Italy; ^2^Dipartimento di Medicina Clinica e Chirurgia, Federico II University Medical School of Naples, Via Sergio Pansini 5, 80131 Naples, Italy; ^3^IRCCS SDN, Napoli Via Gianturco 113, 80143 Naples, Italy; ^4^Dipartimento di Medicina Clinica e Chirurgia, Unit of Endocrinology, Federico II University Medical School of Naples, Via Sergio Pansini 5, 80131 Naples, Italy

## Abstract

**Purpose:**

This study was aimed at evaluating sirtuin 4 (Sirt4) levels in obese individuals, in relation to their adherence to the Mediterranean diet (MD), a healthy dietary pattern characterized by high antioxidant capacity, and markers of visceral fat storage.

**Subjects/Methods:**

Forty-three obese patients (44% males; BMI: 36.7–58.8 kg/m^2^) were consecutively included. PREvención con DIeta MEDiterránea (PREDIMED) and the 7-day food records were used to assess the adherence to MD and dietary pattern, respectively. Visceral adiposity index (VAI) was calculated. Sirt4 levels were detected by ELISA method.

**Results:**

The majority of the obese participants (62.8%) had an average adherence to MD. Compared with average adherers, low adherers had higher BMI, energy intake, and percentage of energy from lipids, mainly saturated fat and polyunsaturated fatty acids (PUFA), and lower Sirt4 levels. After adjusting for BMI, Sirt4 levels remained negatively correlated with VAI. After adjusting for total energy intake, Sirt4 levels remained negatively associated with PREDIMED and consumption of n-3 PUFA, vitamins C and E. The threshold value of PREDIMED predicting the lowest decrease in Sirt4 levels was found at a score of 6.

**Conclusions:**

Less reduced Sirt4 levels in obese patients adhering to MD suggest a further aspect of the antioxidant advantage of MD.

## 1. Introduction

Diet, a modifiable environmental factor, is a mainstay in the management of obese individuals. Mediterranean diet (MD) is a healthy dietary pattern based on a common dietary pattern of the Mediterranean countries [1]. In particular, the MD is characterized by very low amounts of saturated fatty acid (SFA) and a high consumption of polyunsaturated fatty acid (PUFA) and micronutrients, including dietary vitamins and minerals, commonly reported to increase the plasma antioxidant capacity [2]. In addition, the quantity and quality of dietary lipids are known to have an impact on excess storage of lipid as triglycerides (TG) in both adipose tissue and in nonadipose tissue sites, such as the liver [3], which can have subsequent deleterious metabolic effects. On the other hand, the excess storage of lipid occurs as a consequence of continuous oversupply of fatty acids caused by enhanced lipolysis or adipocyte dysfunction, together with an alteration of the fatty acid oxidation in the mitochondria and the production of the reactive oxygen species (ROS), a by-product of oxidative phosphorylation [4, 5]. In particular, an altered fatty acid oxidation pathway plays a key role in the development of hepatic steatosis (HS) and inflammation on a high-fat diet [6]. The visceral adiposity index (VAI) is a gender-specific mathematical index based on simple anthropometric [body mass index (BMI) and waist circumference (WC)] and metabolic [TG and high-density lipoprotein (HDL) cholesterol] parameters [7]. VAI has been proposed as a surrogate marker of adipose tissue distribution and/or dysfunction, independently correlated with insulin sensitivity and cardiometabolic risk both in the general population [8] and in patients with different pathologic conditions, including HS [9].

Sirtuins (Sirt), generally known as lysine deacetylases, are a complex of proteins involved in the control of several biological processes, including lipid oxidative metabolism [10], via the regulation of proteins' acetylation-deacetylation pathways. In turn, Sirt pathways, acting as metabolic sensors in situations of energy stress and nutrient deprivation, are regulated by macronutrient availability [11]. Despite conserved deacetylase domains, mitochondrial sirtuin 4 (Sirt4) is the most efficient lipoamidase among mitochondrial sirtuins. Sirt4 regulates the pyruvate dehydrogenase complex via enzymatic hydrolysis of the lipoamide cofactor, thus modulating acetyl coenzyme A production, Krebs cycle activity, and ROS generation [12]. Afterwards, it became apparent that Sirt4 is involved in the regulation of fatty acid oxidation in the liver [13–15]. In line with this finding, we have reported that circulating levels of Sirt4 were reduced in obese patients with HS, although the possible contribution of the diet and the adipocyte dysfunction on this association was not investigated [16].

The current study is based on the hypothesis that the antioxidant properties of MD might be associated with the circulating levels of Sirt4 in the obese individuals. To clarify these associations, we investigated the possible relationship between circulating levels of Sirt4 and as follows: (i) the adherence to the MD; (ii) the single dietary components evaluated by 7-day food records; and (iii) degree of HS, measured by liver ultrasound and VAI, as marker of adipocyte dysfunction.

## 2. Subjects and Methods

### 2.1. Design and Setting

This is a cross-sectional observational study carried out by the *Endocrinology Unit* of the Department of Clinical Medicine and Surgery at University Federico II in Naples (Italy). The study has been performed in accordance with the Code of Ethics of the World Medical Association (Declaration of Helsinki) for experiments involving humans, and it has been approved by the Ethical Committee of the University Federico II (n.5/14). The purpose of the study was explained to both the patients and the healthy controls, and written informed consent was obtained. The study was conducted without any sponsorship. This cross-sectional observational study was registered at clinicaltrials.gov (NCT02840968).

### 2.2. Population Study

**Figure 1:**
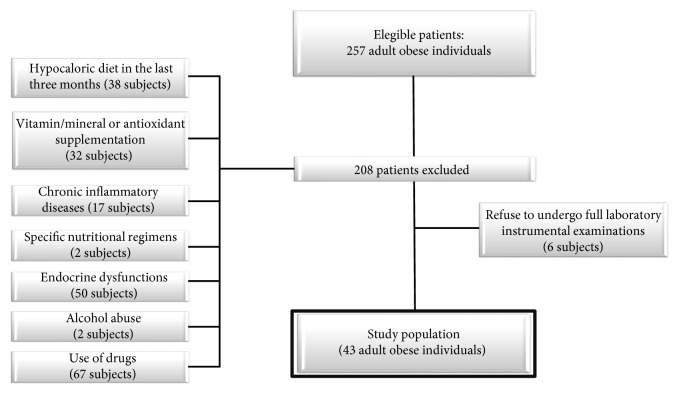
Flow chart of the study design.

Out of the 257 consecutive obese subjects visiting the outpatient Obesity Unit of the Section of *Endocrinology*, University of Naples Federico II, from December 2013 to September 2015, 43 adult individuals referred to our unit for bariatric surgery evaluation and were enrolled in this cross-sectional observational study. A full medical history, including drug use, was collected. Criteria for exclusion from the study were as follows: (a) hypocaloric diet in the last three months or specific nutritional regimens, including vegan or vegetarian diets (38 and two subjects, resp.); (b) the presence of liver or renal failure, cancer, and acute or chronic inflammatory diseases based on a complete medical examination and laboratory investigations (seven subjects with psoriasis, three with rheumatoid arthritis, and seven with chronic obstructive pulmonary disease); (c) occasional or current use of drugs affecting *β*-oxidation, including nonsteroidal anti-inflammatory drugs (18 subjects), *α*-lipoic acid (two subjects), and valproic acid (three subjects); (d) use of weight loss medication (21 subjects) or lipid-lowering drugs (23 subjects); (e) altered thyroid hormone function tests or thyroid hormone treatment or altered somatotropic axis (50 subjects); (f) alcohol abuse according to the Diagnostic and Statistical Manual of Mental Disorders- (DSM-) V diagnostic criteria (two subjects); and (g) vitamin/mineral or antioxidant supplementation (32 subjects). Finally, six subjects dropped out from the study since they refused to undergo full laboratory-instrumental examinations (Figure 1).

Information on smoking habit and physical activity was obtained by a standard questionnaire. Subjects smoking at least one cigarette per day were considered current smokers, while former smokers were the subjects who stopped smoking at least one year before the interview. Remaining participants were defined as noncurrent smokers. Physical activity levels were expressed according to whether the participant habitually engaged at least 30 min/day of aerobic exercise (YES/NO). Physical active participants were advised to avoid the physical activity in the 3 days before blood sampling. Participants were classified according to their alcohol intake into two groups: YES or NO alcohol consumption. Among alcohol consumers, heavy alcohol consumption was defined as more than two standard drinks per day, which is equal to a daily intake of <30 g of alcohol for men and <20 g of alcohol for women.

In all individuals, systolic (SBP) and diastolic (DBP) blood pressure were measured in three times, two minutes apart, with a random zero sphygmomanometer (Gelman Hawksley Ltd., Sussex, UK) after the subject had been sitting for at least 10 min. The average of the second and third reading was recorded.

Hypertension was defined as SBP ≥ 140 mmHg or DBP ≥ 90 mmHg on two different occasions or taking antihypertensive medication. Hypercholesterolemia was defined as a fasting blood total cholesterol level ≥ 190 mg/dL or use of lipid-lowering medication, hypertriglyceridaemia as fasting blood TG levels ≥ 150 mg/dL or use of lipid-lowering medication, and low HDL cholesterol was defined as <40 mg/dL in men and <50 mg/dL in women [17]. A history of using oral hypoglycemic agents or a type 2 diabetes was diagnosed according to American Diabetes Association (ADA) criteria [18].

### 2.3. Anthropometric Measurements

All anthropometric measurements were taken with subjects wearing only light clothes and without shoes. In each subject, BMI was calculated as weight (kg)/height (m^2^). Height was measured to the nearest one cm using a wall-mounted stadiometer. Body weight was determined to the nearest 100 g using a calibrated balance beam scale. The degree of obesity was established on the basis of BMI cut-off points of 30.0–34.9 kg/m^2^ (class I obesity), 35.0–39.9 kg/m^2^ (class II obesity), and ≥40.0 kg/m^2^ (class III obesity) [19]. WC was measured to the closest 0.1 cm with a nonextensible tape. The measurements were made with the subject standing upright, feet together, and arms hanging freely at the sides, with the subjects standing and breathing normally. WC was measured at the midpoint between the inferior costal margin and the upper iliac crest. According to the National Cholesterol Education Program's Adult Treatment Panel III (NCEP-ATP III) criteria, abdominal obesity was defined as WC ≥ 102 cm in men and ≥88 cm in women [20].

### 2.4. Adherence to the Mediterranean Diet

The adherence to the MD was evaluated using the previously validated 14-item questionnaire for the assessment of PREvención con DIeta MEDiterránea (PREDIMED) [21]. A qualified nutritionist administered the questionnaire during a face-to-face interview to all the enrolled subjects. Briefly, for each item, scores 1 and 0 were assigned; PREDIMED score was calculated as follows: 0–5, lowest adherence; score 6–9, average adherence; and score ≥ 10, highest adherence [21].

### 2.5. Dietary Assessment

As already reported by [22], data were obtained during a face-to-face interview between the patient and a qualified nutritionist. In detail, the dietary interview allowed to quantify foods and drinks by using a photographic food atlas (≈1000 photographs) of known portion sizes to ensure accurate completion of the records [23]. Moreover, dietary data, including beverage intakes and alcohol consumption, were collected by a 7-day food records. The subjects returned the records to the nutritionist who asked supplemental questions if necessary. Data were stored and processed using a commercial software (Terapia Alimentare Dietosystem® DS-Medica, http://www.dsmedica.info). Considering quantities and qualities of foods consumed, the software is able to calculate not only the daily caloric intake but also the quantities of macronutrients (protein; total, complex, and simple carbohydrates; total fat, SFA, monounsaturated fatty acids (MUFA), PUFA: n-6 PUFA, n-3 PUFA, and n-6/n-3 PUFAs ratio; cholesterol; and fibers) and antioxidant nutrient intake, such as vitamins A, C, D, and E, and iron, selenium, and zinc. Using the same commercial software, we calculated also the Mediterranean adequacy index (MAI), obtained by dividing the sum of the percentages of dietary energy from food groups typical of a healthy reference MD, by the sum of the percentages of dietary energy of food groups that are not characteristic of a healthy reference MD [24]:

(1) $$\begin{array}{c} MAI=\frac{\left(\%en\;cereals+legumes+potatoes+vegetables+fruit\;fresh\;and\;dry+fish+wine+virgin\;olive\;oil\right)}{\left(\%en\;milk+cheese+meat+eggs+animal\;fats\;and\;margarines+sweet\;beverages+cakes/pies+cookies\right)}. \end{array}$$

The quality was assessed using the Index of Nutritional Quality (INQ), which is based on the nutritional density per 1000 kcal. The INQ was calculated by dividing the nutritional intake per 1000 kcal of total energy intake by the recommended intake (RI) of protein, carbohydrate, and fat per 1000 kcal. The RI values were obtained from the MD recommended, in particular, the macronutrient energy recognized as adequate in the MD is 55–60% of carbohydrates, 10–15% of proteins, and 25–30% fat [25]. Each INQ value ≥ 1.0 indicates adequate nutritional intake, while values < 1.0 indicated inadequate nutritional status. In this study, the INQ was calculated for protein, carbohydrate, and fat [26].

### 2.6. Assay Methods

Samples were collected in the morning between 8 and 10 a.m., after an overnight fast of at least 8 h and stored at −80°C until being processed. All biochemical analyses including fasting plasma glucose, total cholesterol, fasting plasma TG, alanine transaminase (ALT), aspartate aminotransferase (AST), and *γ*-glutamyltransferase (*γ*GT) were performed with a Roche Modular Analytics System in the Central Biochemistry Laboratory of our institution. Low-density lipoprotein (LDL) cholesterol and HDL cholesterol were determined by a direct method (homogeneous enzymatic assay for the direct quantitative determination of LDL and HDL cholesterol). Fasting insulin levels were measured by a solid-phase chemiluminescent enzyme immunoassay using a commercially available kits (Immunolite Diagnostic Products Co, Los Angeles, CA). The intra-assay coefficients of variations (CV) were <5.5%.

### 2.7. Metabolic Indices

Homeostasis model assessment-insulin resistance (HoMA-IR) was calculated according to Matthews et al. [27]: a value of HoMA − IR > 2.5 was used as cutoff of insulin resistance. VAI score has been calculated by the following sex-specific formula, with TG levels expressed in mmol/L and HDL levels expressed in mmol/L:

(2) $$\begin{array}{c} Males:VAI=\left(\frac{WC}{39.68+\left(1.88\times BMI\right)}\right)\times \left(\frac{TG}{1.03}\right)\times \left(\frac{1.31}{HDL}\right),\\ Females:VAI=\left(\frac{WC}{36.58+\left(1.89\times BMI\right)}\right)\times \left(\frac{TG}{0.81}\right)\times \left(\frac{1.52}{HDL}\right). \end{array}$$

Age-specific VAI cut-off values were used according to Amato et al. [8, 28]. In details, cutoffs in subjects aged ≤30, 31–42, 43–52, and 53–66 years were 2.52, 2.23, 1.92, 1.93, respectively [8, 28].

### 2.8. Determination of Circulating Levels of Sirt4

For the determination of circulating levels of Sirt4, venous blood samples from the antecubital vein were obtained following standard procedures. Blood was drawn into a 9 mL serum tube. Samples were centrifuged for 10 min at relative centrifugal force (RCF) of 850–1000. A part of sera was collected, aliquoted, and frozen at −20°C for the successive determination of circulating levels of Sirt4. The kit for in vitro quantitative measurement of Sirt4 in human tissue homogenates and other biological fluids, based on a sandwich enzyme immunoassay, was provided by Uscn Life Science & Technology Company, 3603 Double Lake Dr, Missouri City, TX 77459. The detection range was 0.156–10 ng/mL, as reported elsewhere [16]. Briefly, the standard curve concentrations used for the ELISA were 10 ng/mL, 5 ng/mL, 2.5 ng/mL, 1.25 ng/mL, 0.625 ng/mL, 0.312 ng/mL, and 0.156 ng/mL. The sensitivity of this assay was defined as the lowest protein concentration that could be differentiated from zero. It was determined by adding two standard deviations to the mean optical density value of twenty zero standard replicates and calculating the corresponding concentration. The minimum detectable dose of human Sirt4 was less than 0.051 ng/mL. No significant cross-reactivity or interference between human Sirt4 and other sirtuins was observed, according to manufacturer data. Intra-assay precision was determined as follows: three samples with low, middle, and high level human Sirt4 were tested 20 times on one plate. The inter-assay precision was weighed testing three samples with low, middle, and high level human Sirt4 on 3 different plates, 8 replicates in each plate. The coefficient of variation, calculated by SD/mean × 100 for the intra-assay and inter-assay was <10% and <12%, respectively.

### 2.9. HS Evaluation

The presence of HS, commonly known as “bright liver”, was assessed with a US diagnostic system (Logiq P5, General Electric, Milan, Italy) with a 3.5 MHz convex probe. All the US determinations were made by the same trained operator. The intraoperator variability, as evaluated in 20 subjects within 1 week from the first ultrasonographic examination, showed an overall *r* value of 0.92 (*p* < 0.001).

The classification of “bright liver” or HS was based on the following scale of hyperechogenicity: grade 0 = absent, grade 1 = light, grade 2 = moderate, and grade 3 = severe, pointing out the difference between the densities of the liver and the right kidney [29]. Technically, echo intensity can be influenced by many factors, particularly by gain intensity. To avoid confounding factors that could modify echo intensity and thus bias comparisons, mean brightness levels of both liver and right kidney cortex were obtained on the same longitudinal sonographic plane.

### 2.10. Statistical Methods

The minimum required total sample was calculated using the pooled standard deviation with the level test 0.05 and power 90% of means the circulating levels of Sirt4 in obese patients. With a type I error of 0.10 and a type of II (*β*) error of 0.05, the resulting size was 13 subjects.

Results are expressed as mean ± SD or as median plus range according to the variable's distributions evaluated by Kolmogorov-Smirnov test. To correct for skewed distributions, circulating levels of Sirt4 were logarithmically transformed and back-transformed for presentation in tables and figures.

Differences between two groups were analyzed by Mann–Whitney *U* test or Student's unpaired *t*-test, when appropriate. Differences among three groups were analyzed by the Kruskal-Wallis rank test or ANOVA test, with the Bonferroni test as post hoc test, according to the variable's distribution. The chi^2^ (*χ*^2^) test was used to test the significance of differences in frequency distributions. The correlations between variables were performed using Spearman's *rho* correlation coefficients. Multinomial logistic regression was performed to model the relationship between the circulating levels of Sirt4 and the three groups of HS classification (grade 1, grade 2, and grade 3).

Bivariate proportional odds ratio (OR) models and 95% interval confidence (IC) were performed to assess the association among quantitative variables (all food items of the PREDIMED questionnaire and PREDIMED score). A multiple linear regression model with ordinal predictors was designed to identify critical threshold values (*m*), expressed as *R*^2^, beta (*β*), and *t*, of the PREDIMED score at which significant changes occur in the response circulating levels of Sirt4, as follows:

(3) $$\begin{array}{c} Circulating\;levels\;of\;{Sirt4}_{i}=\beta_{0}+\left(\beta_{1}{PREDIMED\;score}_{i1}+\cdots +\beta_{m-1}{PREDIMED\;score}_{i,m-1}\right)+\varepsilon_{i}. \end{array}$$

For the calculation of ODDS, circulating levels of Sirt4 were dichotomized in two categories, below and above the median (median of circulating levels of Sirt4 was 0.42 ng/mL). The calculation of OR was obtained of bivariate regression analysis model.

In addition, two multiple linear regression analysis models (stepwise method), expressed as *R^2^*, beta (*β*), and *t*, with circulating levels of Sirt4 as dependent variables were used to estimate the predictive value of the following: (a) all food items of PREDIMED questionnaire and PREDIMED score and (b) total energy, SFA, n-3 PUFA, vitamin C, and vitamin E. In these analyses, we entered only those variables that had a *p* value < 0.05 in the univariate analysis (partial correlation). To avoid multicollinearity, variables with a variance inflation factor (VIP) >10 were excluded. Values ≤ 5% were considered statistically significant. Data were stored and analyzed using the MedCalc® package (Version 12.3.0 1993–2012 MedCalc Software bvba-MedCalc Software, Mariakerke, Belgium). Multinomial logistic regression, bivariate proportional OR model, and multiple linear regression model were carried out using the R Project for Statistical Computing 2014 (http://www.R-project.org).

## 3. Results

All participants to the study completed the PREDIMED questionnaires and the 7-day food records. Study population consisted of 43 obese patients, aged 25–53 years (44%, 19 males). BMIs ranged from 36.7 to 58.8 kg/m^2^ (88.4%, 38 subjects were class III–obesity), 27.9% (12 subjects) were hypertensive, and 20.9% (9 subjects) had diagnosis of type 2 diabetes. Median values of HoMA-IR range from 2.5 to 15.7. Median of VAI was 5.3 (1.6–17.3). In particular, VAI was higher than sex and age-specific cutoffs in 79.1% (34 subjects). Moderate/severe HS was diagnosed in 90.7% (39 subjects). Current smokers were 32.6% (14 subjects) and alcohol consumption was reported in 30.2% (13 subjects). A moderate-intensity aerobic activity at least 5 days per week was reported in 25.6% (11 subjects). Among physical active participants, no subject engaged more than 30 min/day/week.

Circulating levels of Sirt4 according to gender, obesity-related comorbidities, metabolic indices, HS, and major lifestyle factors are reported in Table 1. As shown in the table, circulating levels of Sirt4 were significantly different in the presence of obesity-related comorbidities, adiposity dysfunction, HS, and among current smokers or physically inactive individuals, while there were no significant differences according to gender and alcohol consumption.

Response frequency of dietary components included in the PREDIMED questionnaire of the patients is reported in Table 2. Extra virgin olive oil was the most consumed food item, followed by fish with the wine intake in third position. According to the PREDIMED score, all obese participants had a low (PREDIMED score ≤ 5) or average adherence (PREDIMED score 6–9) to the MD, with no subjects reaching a score indicating a high adherence to the MD.

In particular, on the basis of the PREDIMED score, obese participants were divided into low (37.2%) and average adherence (62.8%). Study participants' characteristics grouped according to the PREDIMED score are summarized in Table 3. As shown in table, obese subjects with low adherence to the MD presented with much lower circulating levels of Sirt4, higher BMI, and more altered metabolic indices than average adherers. No differences were observed in age and male-to-female ratio.

In Table 4, we reported the total energy and the daily macronutrients/micronutrients intake obtained from the 7-day food records. As shown in the table, obese individuals with low MD adherence had significantly higher energy intake and percentage of energy from lipids, mainly SFA and PUFA, and a significantly lower fiber intake than average adherers. In addition, low adherers consumed significantly lower complex carbohydrates and antioxidant nutrient intake than average adherers, including n-3 PUFA, A, C, and E vitamins, and micronutrients. In addition, as expected, obese individuals with low MD adherence presented INQ < 1 for protein, carbohydrate, and fat and lower MAI compared with average adherers.

### 3.1. Correlation Studies

The correlations among circulating levels of Sirt4, anthropometric measurements, and metabolic parameters are summarized in Table 5. In Table 6, the multinomial logistic regression of circulating levels of Sirt4 with HS grade are reported. The highest circulating levels of Sirt4 were associated with the lowest odds of HS (grade 1), *p* = 0.002, *R*^2^ = 0.861, and AIC = 56.623.

The results of bivariate proportional odds ratio model performed to assess the association of circulating levels of Sirt4 with food items of PREDIMED questionnaire, and PREDIMED score are reported in Table 7. In obese patients, the lowest decrease in circulating levels of Sirt4 was significantly associated with the highest odds of consumption of the Mediterranean food items, in particular fruits (*p* = 0.002), extra virgin olive oil (*p* = 0.004), vegetables (*p* = 0.008), fish (*p* = 0.011), and wine (*p* = 0.018), and with highest score of adherence of MD (*p* = 0.004). On the contrary, the highest decrease in circulating levels of Sirt4 was significantly associated with the highest odds of consumption of butter (*p* = 0.015). In Table 8, the correlations among circulating levels of Sirt4, PREDIMED score, total energy and daily macronutrients/micronutrients intake, INQ, and MAI evaluated by using the 7-day food records are reported. As expected, INQ and MAI showed a highly positive correlation with PREDIMED score (INQ protein, *r* = 0.793; INQ carbohydrates, *r* = 0.824; INQ fat, *r* = 0.846; MAI, *r* = 0.974; *p* < 0.001, respectively). Circulating levels of Sirt4 correlated with PREDIMED score and MAI (*p* < 0.001), with all the dietary macronutrients and micronutrients, except for total carbohydrates and PUFA (*p* = 0.430) and INQ for each macronutrient (*p* < 0.001). After adjusting for total energy intake, all the associations were lost, except for PREDIMED score (*p* = 0.021), INQ protein (*p* = 0.003), the consumption of fat SFA (*p* = 0.016) and n-3 PUFA (*p* < 0.001), and the intake of vitamins C and E (*p* < 0.001 and *p* = 0.034, respectively); as shown in Table 8. To compare the relative predictive power of the food items included in the PREDIMED and the score of adherence to the MD associated with the circulating levels of Sirt4, we performed a multiple linear regression analysis using models that included as measures of the consumption frequency of each food items of PREDIMED questionnaire along with the PREDIMED score. Using these models, PREDIMED score entered at the first step (*p* < 0.001) and followed by the fruit consumption ≥ 3 serving/day (*p* = 0.009). To compare the relative predictive power of the intake of food and beverage associated with the circulating levels of Sirt4, we performed a multiple linear regression analysis using models that included as measures of the intake of the single micro and macronutrients obtained by the 7-day food records. Using these models, n-3 PUFA entered at the first step (*p* < 0.001), followed by vitamin E (*p* < 0.001) and vitamin C (*p* = 0.048); results are reported in Table 9.

**Figure 2:**
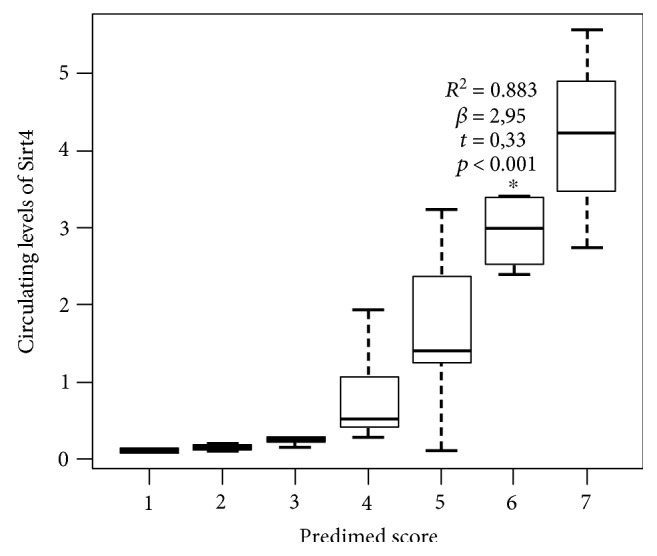
Box plot of value of the ordinal predictors between PREDIMED score and circulating levels of Sirt4. Based on the multiple linear regression analysis with ordinal predictors, the most significant (*α* = 0.05) threshold value of the PREDIMED score predicting the lowest decrease in circulating levels of Sirt4 was found at a score of 6 (^∗^*p* < 0.001). Sirt4: sirtuin 4; PREDIMED: PREvención con DIeta MEDiterránea.

Based on the multiple linear regression analysis with ordinal predictors, the most significant (*α* = 0.05) threshold value of the PREDIMED score predicting the lowest decrease in circulating levels of Sirt4 was found at a score of 6 (*p* < 0.001) (Figure 2).

## 4. Discussion

The present study evidenced a positive association between adherence to the MD and circulating levels of Sirt4 in obese individuals. In addition, a careful investigation of the dietary pattern using the 7-day food records demonstrated that the circulating levels of Sirt4 are negatively correlated with energy intake, and positively with the intake of antioxidant vitamins and minerals considered in this study. Finally, we found that circulating levels of Sirt4 were negatively correlated with surrogate markers of ectopic fat storage and visceral fat dysfunction, independently of the severity of the obesity. Of interest, although experimental studies evidenced that physical activity might affect the Sirt4 expression [30], we found that the associations between Sirt4 and variables included in this study remained significant also after adjusting for physical activity.

We previously reported that in obese individuals with higher degree of HS, there were lower circulating levels of Sirt4 [16]. Although in our preliminary study, we hypothesized the involvement of a high calorie intake in reducing circulating levels of Sirt4; the possible influence of the dietary pattern on the circulating levels of Sirt4 was not investigated.

The present study further extends our previous observations as it demonstrates that lower circulating levels of Sirt4 were actually associated with a specific dietary pattern in obese individuals. In particular, the classification of patients according to the degree of adherence to the MD demonstrated that subjects with an average adherence presented a lower reduction in circulating levels of Sirt4 when compared to subjects with a low adherence, independently of age and gender, with a PREDIMED score ≥ 6 predicting the lowest decrease in circulating levels of Sirt4. Next, the study demonstrated that the highest consumption of food items characteristic of the MD, such as fruits, extra virgin olive oil, vegetables, and fish, was associated with a lowest decrease in circulating levels of Sirt4. The positive association between adherence to the MD and circulating levels of Sirt4 is consistent with the diet-induced modifications in Sirt1 [31] in the larger context of the diet-induced, epigenetic mechanisms [32]. This observation could be of potential interest considering the emerging role of Sirt4 as tumour suppressor recently reported in different human tumour cell lines [33] and the well-known antitumour properties of the MD [34, 35].

The 7-day reports allowed us to obtain detailed information on the diet composition in subjects with low and average adherence to the MD. As expected, we found a highly significant correlation between PREDIMED score and MAI, an index developed to assess how close a diet is to the Healthy Reference National Mediterranean Diet [36]. When compared with average adherers, low adherers had a higher total energy intake, higher consumption of fat, mainly SFA and n-6 PUFA, and lower consumption of complex carbohydrate, protein, MUFA, n-3 PUFA, and fiber, and lower INQ for macronutrients. Consequently, low adherers had a low intake of all antioxidants considered in this study. Of interest, after adjusting these results for total energy intake, circulating levels of Sirt4 remained significantly and positively associated only with the consumption of nutrients with the highest antioxidant properties, such as n-3 PUFA and vitamins C and E, and negatively correlated with the consumption of SFA. The positive association of Sirt4 levels with dietary antioxidant intake and the negative association with VAI might be of particular interest considering the involvement of visceral adiposity in low-grade inflammation [7, 8] and the well-described effects of MD in inflammatory gene expression [37]. Using the multivariate analysis model, circulating levels of Sirt4 were well predicted by the PREDIMED score and the consumption n-3 PUFA.

As expected, obese subjects with a low adherence to MD presented worse anthropometric measurements and metabolic profile. In addition, as expected, low adherence to MD was associated to a higher HS grade when compared with subjects with an average adherence, independently of age and gender. Besides HS, this study reported a novel association between adherence to the MD and circulating levels of Sirt4 with VAI, a well validated, reliable, and widely used marker of ectopic visceral distribution and function [28]. In particular, VAI was higher in low adherers compared with average adherers and showed a negative correlation with circulating levels of Sirt4.

Taking together the results of this study: (i) lend support to the hypothesis that the increase in energy intake in obese individuals was associated with blunted circulating levels of Sirt4 as an adaptive mechanism to decrease fat oxidative capacity, but promoting lipid storage in ectopic tissues; (ii) suggest that the healthy combination of antioxidant vitamins and minerals characteristic of the MD could contribute to dampen this mechanism, probably reducing the oxidative damage and the ectopic fat storage. In line with this hypothesis, circulating levels of Sirt4 were well predicted by either the adherence to the MD and the intake of specific nutrients with antioxidant properties, independently of the energy intake; (iii) let us hypothesize that circulating levels of Sirt4, similarly to Sirt1, might undergo diet-induced epigenetic modifications, thus contributing to the antitumour properties of healthy diets, such as MD; and (iv) evidence that the lowest circulating levels of Sirt4 were associated not only to the highest grade of HS but also to the highest visceral fat dysfunction, independently of the severity of the obesity and physical activity.

Despite these very interesting results, the main limitation of this study is that the cross-sectional design does not allow to identify any causal association between the variables included. Second, we are aware that the sample size is small and the BMI range is wide and is not a measurement of body composition [38]. Nevertheless, we found that circulating levels of Sirt4 were negatively correlated to VAI, an index that incorporates also waist circumference, a far more accurate benchmark for visceral adiposity, also independently of BMI. Third, we do not provide experimental data on muscle biopsy sample between tissue expression and circulating levels of Sirt4; thus, our hypothesis of a compensative regulation of Sirt4 by diet remains speculative. Furthermore, the suggested value of the PREDIMED score for the recognition of the lowest decrease in circulating levels of Sirt4 should be viewed with caution until results will be extended to larger patient populations in order to perform an appropriate cross-validation. However, this study has adequate statistical power, the observed differences between low and average adherers were statistically significant, and correlations have been adjusted for BMI. In addition, in order to improve the power of the study, very stringent inclusion criteria have been applied, such as normal thyroid function or, according to our recent observations [39], normal somatotropic axis. Finally, the strength of this study is the usage of the 7-day food records. Although one week may not be representative of their usual dietary habits, this method of dietary assessment is considered as the “gold standard” in validation studies of different types of self-administered food frequency questionnaires, allowing a more accurate measurement of the real dietary and macronutrient intakes compared to those obtained by retrospective food frequency questionnaires [40, 41]. In addition, to reduce the effect of over- or under-reporting of individual foods, nutrient intake was adjusted for total energy intake.

Thus, although the results were partially limited due to the lack of specific markers of oxidative stress, the detailed information on the diet composition obtained by the 7-day food records was consistent with a more reduced intake of antioxidant nutrients in low adherers compared with average adherers.

### 4.1. Conclusions

A lower reduction in circulating levels of Sirt4 was found in obese individuals with average adherence to the MD compared with low adherers, in association with lower ectopic fat storage and adipocyte dysfunction, independently of BMI. Circulating levels of Sirt4 varied in association not only with energy intake but also with antioxidant nutrient intake. The positive association between the adherence to the MD and circulating levels of Sirt4 suggests that the Sirt4 increase might represent further aspects of the antioxidant and antitumor advantage in obese individuals of the Mediterranean dietary pattern and support the role of qualified nutritionists as members of integrated multidisciplinary teams in the complex management of obesity.

## Figures and Tables

**Table 1:** Circulating levels of Sirt4 in the study population according to gender, obesity-related morbidities, cardiometabolic indices, HS, and major lifestyle factors.

Parameters		Circulating levels of Sirt4 (ng/mL) *n* = 43	
		Median (min–max)	*p* value
Gender	Males	0.37 (0.10–2.75)	0.406
	Females	0.68 (0.11–5.56)	

Hypertension	Yes	0.15 (0.10–0.42)	**<0.001**
	No	1.22 (0.11–5.56)	

Hypercholesterolaemia	Yes	0.28 (0.11–2.51)	**0.015**
	No	0.24 (0.10–5.56)	

Hypertriglyceridaemia	Yes	0.28 (0.11–2.39)	**0.018**
	No	1.30 (0.10–5.56)	

HDL cholesterol	<cutoff	0.18 (0.10–1.26)	**<0.001**
	>cutoff	1.88 (0.16–5.56)	

Type 2 diabetes	Yes	0.15 (0.10–1.11)	**0.001**
	No	0.97 (0.11–5.56)	

VAI	>cutoff	0.29 (0.10–2.66)	**<0.001**
	<cutoff	3.23 (1.22–5.56)	

HS	Grade 1 (mild)	3.79 (1.33–5.56)	
	Grade 2 (moderate)	2.80 (1.66–3.42)	**0.007**
	Grade 3 (severe)	0.24 (0.18–0.37)	

Current smokers	Yes	0.16 (0.10–1.22)	**0.001**
	No	1.26 (0.11–5.56)	

Physical activity	No	0.29 (0.10–2.51)	**0.001**
	Yes	2.75 (0.11–5.56)	

Alcohol consumption	Yes	1.94 (0.10–3.42)	0.190
	No	0.38 (0.11–5.56)	

Circulating levels of Sirt4 were lower in individuals with obesity-related comorbidities, adiposity dysfunction, HS, current smokers, and physically inactive individuals. As HoMA-IR and FLI were increased in all the study population, these variables were not included in this analysis. Circulating levels of Sirt4 were significantly different in the presence of obesity-related comorbidities, adiposity dysfunction, HS, and among current smokers or physically inactive individuals, while there were no significant differences according to gender and alcohol consumption. Results are expressed as median plus range according to variable distributions evaluated by Kolmogorov-Smirnov test. Differences between two groups were analyzed by Mann–Whitney *U* test. Differences among HS grades were analyzed by the Kruskal-Wallis rank test followed by Bonferroni post hoc test. A *p* value in bold type denotes a significant difference (*p* < 0.05). Hypertension was defined as SBP ≥ 140 mmHg or DBP ≥ 90 mmHg on two different occasions or taking antihypertensive medication. Hypercholesterolemia was defined as a fasting blood total cholesterol level ≥ 190 mg/dL or use of lipid-lowering medication, hypertriglyceridaemia was defined as fasting blood triglyceride levels ≥ 150 mg/dL or use of lipid-lowering medication, and low HDL cholesterol was defined as <40 mg/dL in men and <50 mg/dL in women [17]. A history of using oral hypoglycemic agents or a type 2 diabetes was diagnosed according to American Diabetes Association (ADA) criteria [18]. Age-specific VAI cut-off values were used according to Amato et al. [8, 25]. In detail, cutoffs in subjects aged ≤30, 31–42, 43–52, and 53–66 years were 2.52, 2.23, 1.92, and 1.93, respectively [8, 25]. The classification of “bright liver” or HS was based on the following scale of hyperechogenicity: grade 0 = absent, grade 1 = light, grade 2 = moderate, and grade 3 = severe, pointing out the difference between the densities of the liver and the right kidney [26]. Subjects smoking at least one cigarette per day were considered current smokers. Physical activity levels were expressed according to whether the participant habitually engaged at least 30 min/day of aerobic exercise. Among alcohol consumers, heavy alcohol consumption was defined as more than two standard drinks per day, which is equal to a daily intake of <30 g of alcohol for men and <20 g of alcohol for women. Sirt4: sirtuin 4; HDL: high-density lipoprotein; VAI: visceral adiposity index; HS: hepatic steatosis.

**Table 2:** Response frequency of dietary components included in the PREDIMED questionnaire of the patients.

Questions in PREDIMED questionnaire	*n*	%
(1) Use of extra virgin olive oil as main culinary lipid	33	76.7
(2) Extra virgin olive oil > 4 tablespoons	22	51.2
(3) Vegetables ≥ 2 servings/day	21	48.8
(4) Fruits ≥ 3 servings/day	18	41.9
(5) Red/processed meats < 1/day	21	48.8
(6) Butter, cream, margarine < 1/day	19	44.2
(7) Soda drinks < 1/day	10	23.3
(8) Wine glasses ≥ 7/week	24	55.8
(9) Legumes ≥ 3/week	21	48.8
(10) Fish/seafood ≥ 3/week	25	58.1
(11) Commercial sweets and confectionery ≤ 2/week	12	27.9
(12) Tree nuts ≥ 3/week	13	30.2
(13) Poultry more than red meats	21	48.8
(14) Use of sofrito sauce ≥ 2/week	23	53.5

Extra virgin olive oil was the most consumed food item, followed by fish and wine intake. Results are expressed as percentage of response obtained with PREDIMED questionnaire. PREDIMED: PREvención con DIetaMEDiterránea ([20] in the text).

**Table 3:** Sociodemographic and anthropometric characteristics, metabolic profile, and circulating levels of Sirt4 in obese individuals grouped on the basis of the adherence to the Mediterranean diet.

Parameters	Obese Low adherence MD *n* = 16	Obese Average adherence MD *n* = 27	*p* value
Age (years)	38.1 ± 3.4	35.9 ± 7.7	0.356
Gender M/F	8/8	11/16	0.784
*Anthropometric measurement*			
BMI (kg/m^2^)	52.0 ± 3.4	42.4 ± 2.9	**<0.001**
WC males (cm)	151.1 ± 9.8	136.8 ± 11.6	**0.012**
WC females (cm)	144.4 ± 17.1	125.4 ± 15.4	**0.012**
*Metabolic profile*			
Circulating levels of Sirt4 (ng/mL)	0.16 (0.10–0.29)	1.33 (0.11–5.56)	**<0.001**
SBP (mmHg)	136.0 ± 12.4	127.4 ± 10.7	**0.022**
DBP (mmHg)	85.0 (70.0–100.0)	80.0 (60.0–95.0)	0.090
Fasting glucose (mg/dL)	109.0 (73.0–143.0)	94.0 (68.0–193.0)	**0.004**
Insulin (*μ*U/mL)	36.1 ± 7.9	26.7 ± 8.5	**0.001**
Total cholesterol (mg/dL)	211.1 ± 31.9	179.4 ± 27.4	**0.003**
HDL cholesterol (mg/dL)	36.8 ± 6.1	51.3 ± 11.1	**<0.001**
LDL cholesterol (mg/dL)	134.9 ± 23.0	102.8 ± 26.5	**<0.001**
Triglycerides (mg/dL)	171.5 (89.0–380.0)	101.0 (49.0–283.0)	**0.015**
ALT (U/L)	38.0 (12.0–91.0)	32.0 (13.0–99.0)	0.439
AST (U/L)	25.0 (12.0–52.0)	23.0 (10.0–91.0)	0.793
*γ*GT (U/L)	30.0 (12.0–61.0)	30.5 (14.0–101.0)	0.776
*Metabolic indices*			
HoMA-IR	10.0 ± 3.0	6.4 ± 2.4	**<0.001**
VAI	8.5 (3.3–17.3)	4.7 (1.6–15.8)	**0.003**
*HS evaluation*			
HS (grade 1/2/3)	0/1/15	4/9/14	**<0.001**
FLI	99.9 (98.8–100.0)	98.7 (80.3–99.9)	**<0.001**

Obese individuals with low adherence to the MD exhibited statistically significant differences in anthropometric measurements, metabolic profile, metabolic indices, and HS evaluation compared with average adherer counterpart. No differences were observed in age and male-to-female ratio. Results are expressed as mean ± standard deviation or as median plus range according to variable distributions evaluated by Kolmogorov–Smirnov test. Differences between groups were analyzed by unpaired Student's *t*-test or Wilcoxon signed-rank test, when appropriate. The chi^2^ (*χ*^2^) test was used to test the significance of differences between the two groups. *p* value indicates comparison between those with low adherence versus those with average adherence to the MD. A *p* value in bold type denotes a significant difference (*p* < 0.05). MD: Mediterranean diet; BMI: body mass index; WC: waist circumference; Sirt4: sirtuin 4; SBP: systolic blood pressure; DBP: diastolic blood pressure; HDL: high-density lipoprotein; LDL: low-density lipoprotein; ALT: alanine aminotransferase; AST: aspartate aminotransferase; *γ*GT: gamma glutamyl transferase: HoMA-IR: homeostatic model assessment-insulin resistance; VAI: visceral adiposity index; HS: hepatic steatosis; FLI: fatty liver index.

**Table 4:** Total energy and daily macronutrient/micronutrient intake of obese individuals grouped on the basis of the adherence to the Mediterranean diet.

Parameters	Obese Low adherence MD *n* = 16	Obese Average adherence MD *n* = 27	*p* value
Total energy (kcal)	3473.1 ± 271.9	2822.3 ± 287.7	**<0.001**
Protein (% of total kcal)	14.0 (11.0–18.0)	16.0 (12.0–20.0)	**0.045**
INQ protein	0.98 ± 0.03	1.08 ± 0.07	**<0.001**
Carbohydrate (% of total kcal)	56.0 (53.0–61.0)	57.0 (54.0–60.0)	0.469
INQ carbohydrate	0.97 ± 0.03	1.03 ± 0.03	**<0.001**
Complex (% of total kcal)	28.8 ± 5.5	31.8 ± 4.0	**0.049**
Simple (% of total kcal)	27.8 ± 4.5	25.2 ± 4.0	0.055
Fat (% of total kcal)	29.0 (28.0–30.0)	27.0 (25.0–30.0)	**<0.001**
INQ Fat	0.96 ± 0.03	1.06 ± 0.05	**<0.001**
SFA (% of total kcal)	11.5 (8.3–15.7)	9.0 (1.7–10.3)	**<0.001**
MUFA (% of total kcal)	12.8 (11.2–14.3)	15.7 (14.5–17.7)	**<0.001**
PUFA (% of total kcal)	3.3 (1.8-9.3)	2.9 (1.8–5.9)	0.191
n-6 PUFA (g/day)	10.9 (4.8–34.7)	5.7 (0.4–15.4)	**0.001**
n-3 PUFA (g/day)	2.2 (0.6–2.8)	3.4 (1.3–12.4)	**<0.001**
n-6/n-3 PUFAs ratio	5.9 (2.1–20.8)	1.6 (0.1–11.8)	**<0.001**
Cholesterol (mg/day)	335.9 ± 42.2	323.0 ± 32.2	0.262
Fiber (g/day)	20.4 (8.2–25.0)	24.0 (10.1–28.1)	**0.021**
Retinol Eq (*μ*g/day)	756.5 (451.0–1990.0)	1249.0 (527.0–2012.0)	**0.001**
Vitamin C (mg/day)	71.0 ± 26.3	159.0 ± 44.7	**<0.001**
Vitamin D (*μ*g/day)	1.6 ± 0.6	2.1 ± 0.7	**0.014**
Vitamin E (mg/day)	10.5 ± 1.6	16.4 ± 2.3	**<0.001**
Iron (mg/day)	8.2 ± 1.9	10.6 ± 2.9	**0.005**
Selenium (*μ*g/day)	15.2 (9.0–33.9)	22.1 (8.7–42.5)	**0.003**
Zinc (mg/day)	8.8 ± 1.5	10.4 ± 1.3	**<0.001**
MAI	1.09 ± 0.34	3.18 ± 0.81	**<0.001**

Obese individuals with low MD adherence had a higher energy intake, a higher percentage of energy from lipids, mainly SFA and PUFA, and a lower fiber intake than average adherers. Low adherers consumed lower complex carbohydrates and lower antioxidant intake than average adherers, including n-3 PUFA, A, C, and E vitamins, and micronutrients. Results are expressed as mean ± standard deviation or as median plus range according to variable distributions evaluated by Kolmogorov-Smirnov test. Differences between groups were analyzed by unpaired Student's *t*-test or Wilcoxon signed-rank test, when appropriate. *p* value indicates comparison between those with low adherence versus those with average adherence to the MD. A *p* value in bold type denotes a significant difference (*p* < 0.05). MD: Mediterranean diet; INQ: index of nutritional quality; SFA: saturated fatty acids; MUFA: monounsaturated fatty acids; PUFA: polyunsaturated fatty acids; MAI: Mediterranean adequacy index.

**Table 5:** Correlations among circulating levels of Sirt4 with demographics, anthropometric measurements, metabolic profile, metabolic indices, and HS evaluation.

Parameters	Circulating levels of Sirt4 (ng/mL) *n* = 43			
	Simple correlation		Adjusted for BMI	
	*r*	*p* value	*r*	*p* value
Age (years)	−0.363	0.017	−0.249	0.122
*Anthropometric measurement*
BMI (kg/m^2^)	−0.804	**<0.001**	—	**—**
WC (cm)	−0.707	**<0.001**	−0.339	**0.033**
*Metabolic profile*
SBP (mmHg)	−0.512	**<0.001**	0.038	0.817
DBP (mmHg)	−0.318	0.038	0.075	0.645
Fasting glucose (mg/dL)	−0.369	0.015	−0.067	0.679
Insulin (*μ*U/mL)	−0.543	**<0.001**	−0.147	0.366
Total cholesterol (mg/dL)	−0.522	**<0.001**	−0.257	0.109
HDL cholesterol (mg/dL)	0.814	**<0.001**	0.434	**0.005**
LDL cholesterol (mg/dL)	−0.642	**<0.001**	−0.276	0.085
Triglycerides (mg/dL)	−0.454	**0.002**	−0.246	0.126
ALT (U/L)	0.081	0.611	0.130	0.424
AST (U/L)	-0.015	0.926	0.041	0.801
*γ*GT (U/L)	0.117	0.468	−0.006	0.971
*Metabolic indices*
HoMA-IR	−0.627	**<0.001**	−0.111	0.497
VAI	-0.559	**<0.001**	−0.329	**0.038**
*HS evaluation*
FLI	−0.762	**<0.001**	−0.633	**<0.001**

Correlations among variables were performed using Spearman's *rho* correlation coefficients. Circulating levels of Sirt4 were significantly associated with BMI, WC, metabolic profile, and the metabolic indices. After adjusting for BMI, the associations between circulating levels of Sirt4 and the study variables were not significant, except for WC, HDL cholesterol, VAI, and FLI. A *p* value in bold type denotes a significant difference (*p* < 0.05). Sirt4: sirtuin 4; BMI: body mass index; WC: waist circumference; SBP: systolic blood pressure; DBP: diastolic blood pressure; HDL: high-density lipoprotein; LDL: low-density lipoprotein; ALT: alanine aminotransferase; AST: aspartate aminotransferase; *γ*GT: gamma glutamyl transferase; HoMA-IR: homeostatic model assessment-insulin resistance; VAI: visceral adiposity index; HS: hepatic steatosis; FLI: fatty liver index.

**Table 6:** Multinomial logistic regression of circulating levels of Sirt4 with HS grade.

HS	OR	*p* value	95% IC
Grade 1 = light	9.47	**0.002**	7.02–12.77
Grade 2 = moderate	3.22	**0.005**	2.39–4.35
Grade 3 = severe	1.00	**0.003**	0.74–1.34

Multinomial logistic regression of circulating levels of Sirt4 with HS grade. The highest levels of Sirt4 were associated with the lowest odds of HS (grade 1). A *p* value in bold type denotes a significant difference (*p* < 0.05). Sirt4: sirtuin 4; HS: hepatic steatosis; OR: odds ratio; IC: interval confidence.

**Table 7:** Bivariate proportional odds ratio model to assess the association between circulating levels of Sirt4 and food items included in the PREDIMED questionnaire.

Questions	OR	*p* value	95% IC	AIC	*R* ^2^adj
(1) *Use of extra virgin olive oil as main culinary lipid*		**0.004**		39.484	0.239
Yes	2.60		1.93–3.51		
No	1.04		0.77–1.40		

(2) *Extra virgin olive oil > 4 tablespoons*		0.234		62.069	0.254
Yes	0.74		0.55–1.00		
No	3.65		2.70–4.92		

(3) *Vegetables ≥ 2 servings/day*		**0.008**		53.157	0.175
Yes	2.61		1.93–3.52		
No	1.03		0.77–1.44		

(4) *Fruits ≥ 3 servings/day*		**0.002**		46.918	0.265
Yes	3.44		2.55–4.63		
No	0.78		0.58–1.06		

(5) *Red/processed meats < 1/day*		0.246		62.15	0.024
Yes	1.33		0.98–1.79		
No	2.04		1.51–2.75		

(6) *Butter, cream, margarine < 1/day*		**0.015**		52.652	0.175
Yes	0.35		0.26–0.43		
No	7.74		5.74–10.44		

(7) *Soda drinks < 1/day*		0.593		50.37	0.058
Yes	1.14		0.85–1.54		
No	2.36		1.75–3.19		

(8) *Wine glasses ≥ 7/week*		**0.018**		54.708	0.140
Yes	2.41		1.78–3.25		
No	1.12		0.83–1.52		

(9) *Legumes ≥ 3/week*		0.067		59.615	0.066
Yes	1.65		1.23–2.23		
No	1.63		1.21–2.20		

(10) *Fish/seafood ≥ 3/week*		**0.011**		50.762	0.200
Yes	3.36		2.49–4.53		
No	0.80		0.59–1.08		

(11) *Commercial sweets and confectionery ≤ 2/week*		0.095		52.011	0.057
Yes	1.53		1.13–2.06		
No	1.77		1.31–2.39		

(12) *Tree nuts ≥ 3/week*		0.220		55.183	0.028
Yes	1.35		1.00–1.82		
No	2.01		1.49–2.71		

(13) *Poultry more than red meats*		0.146		61.239	0.039
Yes	1.45		1.07–1.96		
No	1.86		1.38–2.52		

(14) *Use of sofrito sauce ≥ 2/week*		0.107		60.355	0.051
Yes	1.56		1.15–2.10		
No	1.74		1.29–2.34		

*PREDIMED score*		**0.010**		20.409	0.710
Low adherence MD (score ≤ 5)	3.02		2.24–4.08		
Average adherence MD (6 ≤ score ≤ 9)	8.97		1.21–6.65		

Circulating levels of Sirt4 were associated with the highest odds of consumption fruits, extra virgin olive oil, vegetables, fish, and wine and with highest score of adherence of MD. On the contrary, the highest levels of Sirt4 were significantly associated with the lowest odds of consumption of butter. A *p* value in bold type denotes a significant difference (*p* < 0.05). PREDIMED: PREvención con DIeta MEDiterránea; OR: odds ratio; IC: interval confidence; AIC: Akaike information criterion.

**Table 8:** Correlations of circulating levels of Sirt4 with PREDIMED score, total energy, and daily macronutrient/micronutrient intake.

Parameters	Circulating levels of Sirt4 (ng/mL) *n* = 43			
	Simple correlation		Adjusted for total energy (kcal)	
	*r*	*p* value	*r*	*p* value
PREDIMED score	0.820	**<0.001**	0.356	**0.021**
Total energy (kcal)	−0.818	**<0.001**	—	**—**
Protein (% of total kcal)	0.511	**<0.001**	0.100	0.529
INQ protein	0.742	**<0.001**	0.453	**0.003**
Carbohydrate (% of total kcal)	−0.070	0.656	−0.033	0.838
INQ carbohydrate	0.661	**<0.001**	0.184	0.242
Complex (% of total kcal)	0.395	**0.009**	0.082	0.606
Simple (% of total kcal)	−0.472	**0.001**	−0.133	0.477
Fat (% of total kcal)	−0.822	**<0.001**	−0.280	0.073
INQ Fat	0.578	**<0.001**	0.060	0.704
SFA (% of total kcal)	−0.730	**<0.001**	−0.370	**0.016**
MUFA (% of total kcal)	0.739	**<0.001**	0.138	0.384
PUFA (% of total kcal)	−0.124	0.430	0.050	0.754
n-6 PUFA (g/day)	−0.546	**<0.001**	−0.107	0.502
n-3 PUFA (g/day)	0.854	**<0.001**	0.646	**<0.001**
n-6/n-3 PUFAs ratio	−0.541	**<0.001**	0.123	0.438
Cholesterol (mg/day)	−0.336	**0.028**	−0.007	0.964
Fiber (g/day)	0.305	**0.047**	−0.026	0.872
Retinol Eq (*μ*g/day)	0.337	**0.027**	−0.029	0.854
Vitamin C (mg/day)	0.825	**<0.001**	0.476	**0.001**
Vitamin D (*μ*g/day)	0.407	**0.007**	0.083	0.601
Vitamin E (mg/day)	0.834	**<0.001**	0.329	**0.034**
Iron (mg/day)	0.335	**0.028**	0.067	0.673
Selenium (*μ*g/day)	0.421	**0.005**	0.205	0.192
Zinc (mg/day)	0.505	**0.001**	0.020	0.900
MAI	0.833	**<0.001**	0.435	**0.004**

Correlations among variables were performed Spearman's *rho* correlation coefficients. Circulating levels of Sirt4 were significantly associated with all the macronutrients/micronutrients evaluated in this study, except with carbohydrate and n-6 PUFA. After adjusting for total energy intake, the associations between circulating levels of Sirt4 and the study variables were lost, except for PREDIMED score, the consumption of fat, SFA and n-3 PUFA, and the intake of vitamins C and E. Sirt4: sirtuin 4; PREDIMED: PREvención con DIeta MEDiterránea; INQ: index of nutritional quality; SFA: saturated fatty acids; MUFA: monounsaturated fatty acids; PUFA: polyunsaturated fatty acids; MAI: Mediterranean adequacy index.

**Table 9:** Multiple regression analysis models (stepwise method) with the circulating levels of Sirt4 as dependent variable to estimate the predictive value of the following: (a) food items of PREDIMED questionnaire and PREDIMED score and (b) total energy intake, SFA, n-3 PUFA, vitamin C, and vitamin E.

Parameters	Multiple regression analysis			
*Model 1*	*R* ^2^	*β*	*t*	*p* value
PREDIMED score	0.335	0.579	4.6	**<0.001**
Fruits ≥3 servings/day	0.442	0.368	2.8	**0.009**
*Variable excluded: other items of PREDIMED score*				

*Model 2*	*R* ^2^	*β*	*t*	*p* value
n-3 PUFA	0.729	0.854	10.5	**<0.001**
Vitamin E	0.835	0.461	5.1	**<0.001**
Vitamin C	0.851	0.241	2.0	**0.048**
*Variable excluded: total energy and SFA*				

Among adherence to the MD and food items (model 1) and among dietary macronutrient and micronutrient intake (model 2), circulating levels of Sirt4 were well predicted by PREDIMED score and n-3 PUFA. A *p* value in bold type denotes a significant difference (*p* < 0.05). Sirt4: sirtuin 4; PREDIMED: PREvención con DIeta MEDiterránea; SFA: saturated fatty acids; PUFA: polyunsaturated fatty acids.

## Abbreviations

ALT: Alanine aminotransferase
AST: Aspartate aminotransferase
BMI: Body mass index
DBP: Diastolic blood pressure
HDL: High-density lipoprotein
HoMA-IR: Homeostatic model assessment-insulin resistance
HS: Hepatic steatosis
INQ: Index of nutritional quality
LDL: Low-density lipoprotein
MAI: Mediterranean adequacy index
MD: Mediterranean diet
MUFA: Monounsaturated fatty acids
PREDIMED: PREvención con DIeta MEDiterránea
PUFA: Polyunsaturated fatty acids
RI: Recommended intake
ROS: Reactive oxygen species
SBP: Systolic blood pressure
SFA: Saturated fatty acids
Sirt4: Sirtuin 4
TG: Triglycerides
VAI: Visceral adiposity index
WC: Waist circumference
*γ*GT: Gamma glutamyl transferase.
